# A missense variant in the coil1A domain of the *keratin 25* gene is associated with the dominant curly hair coat trait (Crd) in horse

**DOI:** 10.1186/s12711-017-0359-5

**Published:** 2017-11-15

**Authors:** Caroline Morgenthaler, Mathieu Diribarne, Aurélien Capitan, Rachel Legendre, Romain Saintilan, Maïlys Gilles, Diane Esquerré, Rytis Juras, Anas Khanshour, Laurent Schibler, Gus Cothran

**Affiliations:** 1 UMR1313, Génétique Animale et Biologie Intégrative, INRA, AgroParisTech, Université Paris-Saclay, 78350 Jouy-en-Josas, France; 2 Département R&D, ALLICE, 149 rue de Bercy, 75595 Paris Cedex 12, France; 3 UMR444, Laboratoire de Génétique Cellulaire, INRA, Castanet-Tolosan, 31326 France; 4 Department of Veterinary Integrative Biosciences, College of Veterinary Medicine and Biomedical Sciences, Texas A&M University, College Station, TX 77843 USA; 5 Texas Scottish Rite Hospital for Children, Dallas, TX USA

## Abstract

**Background:**

Curly horses present a variety of curl phenotypes that are associated with various degrees of curliness of coat, mane, tail and ear hairs. Their origin is still a matter of debate and several genetic hypotheses have been formulated to explain the diversity in phenotype, including the combination of autosomal dominant and recessive alleles. Our purpose was to map the autosomal dominant curly hair locus and identify the causal variant using genome-wide association study (GWAS) and whole-genome sequencing approaches.

**Results:**

A GWAS was performed using a Bayesian sparse linear mixed model, based on 51 curly and 19 straight-haired French and North American horses from 13 paternal families genotyped on the Illumina EquineSNP50 BeadChip. A single strong signal was observed on equine chromosome 11, in a region that encompasses the *type I keratin* gene cluster. This region was refined by haplotype analysis to a segment including 36 genes, among which are 10 keratin genes (*KRT*-*10*, -*12*, -*20*, -*23*, -*24*, -*25*, -*26*, -*27*, -*28*, -*222*). To comprehensively identify candidate causal variants within all these genes, whole-genome sequences were obtained for one heterozygous curly stallion and its straight-haired son. Among the four non-synonymous candidate variants identified and validated in the curly region, only variant g.21891160G>A in the *KRT25* gene (KRT25:p.R89H) was in perfect agreement with haplotype status in the whole pedigree. Genetic association was then confirmed by genotyping a larger population consisting of 353 horses. However, five discordant curly horses were observed, which carried neither the variant nor the main haplotype associated with curliness. Sequencing of *KRT25* for two discordant horses did not identify any other deleterious variant, which suggests locus rather than allelic heterogeneity for the curly phenotype.

**Conclusions:**

We identified the KRT25:p.R89H variant as responsible for the dominant curly trait, but a second dominant locus may also be involved in the shape of hairs within North American Curly horses.

**Electronic supplementary material:**

The online version of this article (10.1186/s12711-017-0359-5) contains supplementary material, which is available to authorized users.

## Background

**Fig. 1:**
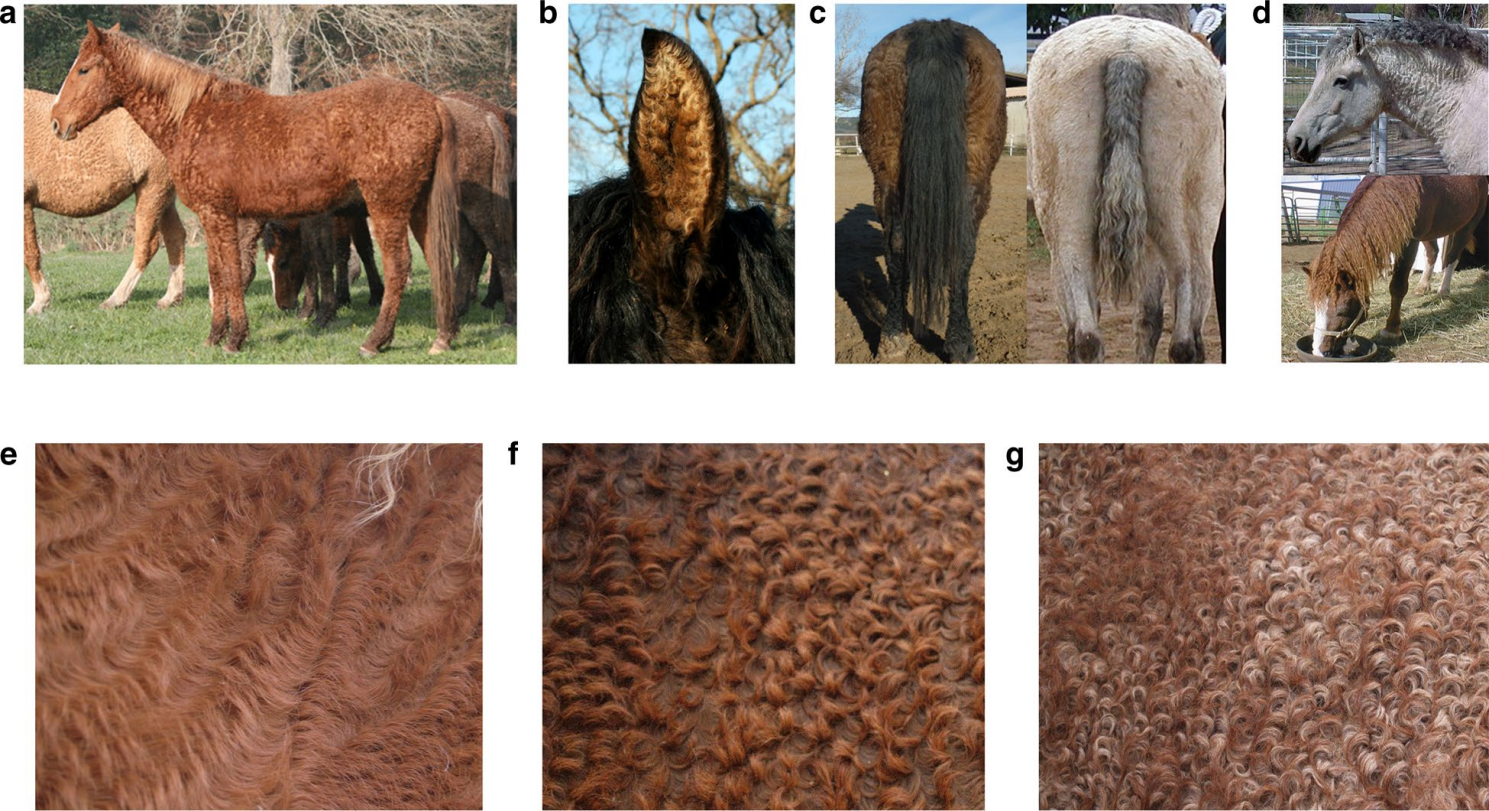
Phenotype of curly horses. Curly horses present curly haired coat (**a**) and ear (**b**). Tail (**c**) and mane (**d**) present phenotypes ranging from curly to wavy hairs and can resemble dreadlocks. The coat phenotype is associated with various degrees of curliness, ranging from slightly curled (**e**) and curled (**f**) up to extremely dense “micro curled” (**g**) hairs. Pictures were from the ICHO association with permission of Dr. Mitch Wilkinson and Bunny Reveglia (see http://www.ichocurlyhorses.com/ and http://ichophotos.weebly.com)

First described by Crow and Sioux Native American tribes as early as 1801 during winter horse counting [1], Curly horses present a variety of coat curl phenotypes associated with diverse degrees of curliness which can range from “minimal” scattered curled hair up to extremely dense “micro curled” permanent coat as observed in certain Missouri Fox Trotter lines (Fig. 1).

Some rare cases of partial alopecia that resemble the rex coat phenotype (e.g. [2]) have also been reported and defined as “extreme”. As expected in hair traits, these various phenotypes fluctuate depending on the season. During summer, pelage is short and the curls appear smooth to slightly wavy while they form tight ringlets to a marcel type wave as hair grows during winter. The mane and tail hairs also present particular phenotypes ranging from curly to wavy or sometimes resembling dreadlocks [3]. In contrast, ear hairs are constantly curly and constitute a good marker to ascertain the phenotype when coat curliness is minimal (Fig. 1). Curly horses are claimed to be hypoallergenic and some studies suggest that regular contact with these horses abolishes the mild allergic reactions of the start period of contact in most allergic riders [4].

The origin of the contemporary North American Curly horses is still a matter of debate. According to the American Bashkir Curly Horse Registry, they are assumed to descend either from Central Asia lineages such as the Lokai breed, imported by fur traders or from Western USA feral horses, possibly with Spanish background [5]. Since several European breeds also exhibit curly hair, Spanish conquistadors may also have brought the curly gene to South America. These hypotheses are not mutually exclusive and the crossbreeding of different lineages may be the source of the genetic and phenotypic heterogeneities reported. Indeed, in North American horses, the most frequent Curly coat phenotype is described as autosomal dominant (Crd) [6], whereas recessive forms have been reported to segregate in the Quarter Horse, Arabian, Appaloosa, Missouri Fox Trotter, Tennessee Walking Horse, Paint, Morgan and Paso Fino breeds [6] as well as in French Percheron horses [7].

Thus, the aim of this study was to map the autosomal dominant Curly locus and identify the causal variant by combining genome-wide association study (GWAS) and whole-genome sequencing approaches.

## Methods

### Ethics statement

Experiments reported in this work comply with the French National Institute for Agricultural Research (INRA) as well as Texas A&M University ethical guidelines.

### Animals, phenotyping and sampling

Two distinct populations were collected to conduct this study. The USA design was primarily focused on curly stallions proved to carry the Crd dominant gene and on their offspring, in order to avoid confounding effects due to the recessive variant (curly stallions producing both curly and straight-haired offspring from both curly and straight haired mares). Likewise, the French design mainly focused on five families, in which the Crd gene segregates and stallions were used to mate both curly and non-curly mares. Phenotypes were obtained from registry records as well as from breeders and veterinarian reports. Animals studied in this project are listed in Additional file 1: Table S1.

One EDTA-blood sample was collected per animal to isolate DNA for genotyping. DNA was prepared by using either the PUREGENE DNA PURIFICATION SYSTEM (Gentra Systems, Minneapolis, MN) in the USA or the DNeasy Blood and Tissue Kit (Qiagen) in France. Samples were analyzed quantitatively and qualitatively by spectrophotometry.

### Genome-wide association study (GWAS)

In France, 48 progeny from 13 paternal families were genotyped with the Illumina single nucleotide polymorphism Equine SNP50 BeadChip by the LABOGENA laboratory using routine procedures (Jouy-en-Josas, France). One curled animal was discarded due to poor DNA quality (call rate lower than 95%). Likewise, 24 horses (12 curly and 12 straight-haired) were genotyped in the USA using the Illumina EquineSNP50 BeadChip by the GeneSeek company. One sample was discarded due to parentage error. Finally, the combined dataset used for GWAS comprised 70 animals (51 curly and 19 straight-haired) (see Additional file 1: Table S1). SNPs with a call frequency lower than 95%, a minor allele frequency lower than 5% and a probability of Hardy‐Weinberg equilibrium less than 0.001 were removed. Finally, only autosomal SNPs with a confirmed position on the horse genome assembly EquCab2.0 were retained [8], resulting in a set of 46,215 SNPs for subsequent analyses. SNP order and map distances were based on the horse genome assembly EquCab2.0. A genome-wide association study was performed using a Bayesian sparse linear mixed model (BSLMM) available in the GEMMA software [9]. This hybrid GWAS analysis approach yields good performance across a wide range of scenarios, including typical case–control GWAS on binary phenotypes. BSLMM controls for population stratification, individual relatedness, or unmeasured confounding factors when performing association tests in genetic association studies. In this model, effects of SNPs are decomposed into two parts: α that captures the small effects that all SNPs have (genetic background), and β the additional large effects of some SNPs (QTL effects). The BSLMM approach involves prior specification for several parameters, which clearly vary across different datasets and have to be estimated. This is done within a Bayesian framework by specifying prior distributions for the parameters, and using Markov chain Monte Carlo (MCMC) simulation to obtain approximate samples from their posterior distribution given the observed data. At each iteration step, a maximum proportion of π SNPs with a non-zero effect is retained, making it possible to define a set of SNPs with the highest probability of being associated with the trait of interest. For this study, a maximum of 140 SNPs were retained at each iteration, corresponding to a π of 0.003, and 1,000,000 iterations were run. For each SNP, the posterior probability of inclusion was computed using the GEMMA software as the frequency of retention of this SNP across all iterations. This posterior inclusion probability can be directly interpreted as the probability that a SNP has an effect above the polygenic background with a certain effect size, which is more intuitive to interpret than a *p* value from a single-SNP analysis. Here, cumulative probabilities were computed for sliding windows of 15 SNPs and plotted using the SNP in the middle of the window as reference.

### Haplotype analysis

Sliding windows with cumulative inclusion probabilities higher than 75% based on the BSLMM analysis were combined to define a mapping interval centered on the most significant SNP (BIEC2-143630). Haplotypes were reconstructed with FImpute software [10] using linkage disequilibrium and pedigree information (over at least four generations). Then, haplotypes were visually examined to identify a putative identical-by-descent segment shared by most of the curly animals and rare or absent in the straight-haired animals. Founder haplotypes were defined as “non-curly” haplotypes carried by crossbreed animals plus the six most frequent haplotypes (see Additional file 1: Table S2a).

### Whole-genome sequencing and screening for the causative variant

Paired-end libraries with a 250-bp insert size were generated for BCF SPARTACULAR SPLASHES, a curly horse that was heterozygous for the IBD haplotype GGAGAGAAAA at the type I keratin gene cluster (see “Results and discussion” section), and ALIAS SPLASH, one of its straight-haired sons, using the Illumina TruSeq DNA Sample Prep kit. The two libraries were quantified using the QPCR Library Quantification kit (Agilent), controlled on a High Sensitivity DNA chip (Agilent) and sequenced on two HiSeq 2000 lanes (Illumina), each with Illumina TruSeq V3 kit (200 cycles). Reads were submitted to the European Nucleotide Archive (ENA, www.ebi.ac.uk/ena) under study accession number PRJEB19479 and run accession numbers ERR2027931 and ERR2027932. The 100-bp reads were mapped on the horse genome assembly EquCab2.0 [8] using the BWA tool [11]. Base quality scores in aligned BAM files were recalibrated using GATK [12]. Only reads with a unique mapping and a minimal quality of 30 were retained. PCR duplicates were filtered and variants were called using SAMtools [13]. Variants that were located within the interval including the Curly locus were annotated using variant effect predictor [14] based on Ensembl Release 78. These variants were then filtered to retain only those, which were (1) present in the heterozygous state in the curly animal, (2) absent from its straight-haired son and (3) unknown in other non-curled breeds based on dbSNP. Likewise, two discordant horses (DRAKVALLMONS ITE O MAGUZU and JAK BOREAL MAGUZU) were sequenced to search for other candidate variants associated with curliness.

### Genotyping of the four non-synonymous variants

Four non-synonymous candidate variants on *Equus caballus* chromosome (ECA) 11 (g.21891160G>A, g.21932167G>T, g.22186465C>T, g.22191762G>T), were genotyped by PCR and Sanger sequencing for at least ten animals that carried the regular curly haplotype and ten non-carriers. Primers were designed from the EquCab2.0 genome assembly with Primer-BLAST [15] and are in Additional file 1: Table S3. PCR reactions were performed using the Go-Taq Flexi (Promega) according to the manufacturer’s instructions on a Mastercycler pro thermocycler (Eppendorf). The resulting amplicons were purified and bidirectionally sequenced by Eurofins MWG (Germany) using conventional Sanger sequencing. Variants were detected with the NovoSNP software [16]. Finally, variant g.21891160G>A (KRT25:p.R89H) was genotyped on an extended panel of 150 curly and 203 randomly chosen straight-haired animals from 35 different breeds using Custom TaqMan allelic discrimination assay on a ABI 7500 Real-Time PCR system (Applied Biosystems) according to the manufacturer’s recommendations. The custom TaqMan SNP Genotyping assay for SNP g.21891160G>A (KRT25:p.R89H) used forward primer 5′-TGAGAAGGTGACCATGCAGAAC-3′, reverse primer 5′-ACGCACATTCTCCAGGTAGGA-3′ as well as mutant probe (FAM) 5′-CAACGACCACCTTGC-3′ and wild-type probe (VIC) 5′-CAACGACCGCCTTGC-3′.

### Alignment of proteins

Proteins were recovered from Ensembl (Ensembl Genome Browser, http://www.ensembl.org) and multiple alignments were performed with full amino acid sequences using CLUSTAL W (http://www.ebi.ac.uk/clustalw/).

## Results and discussion

### The curly hair phenotype maps to the *type I keratin* gene cluster on ECA11

**Fig. 2:**
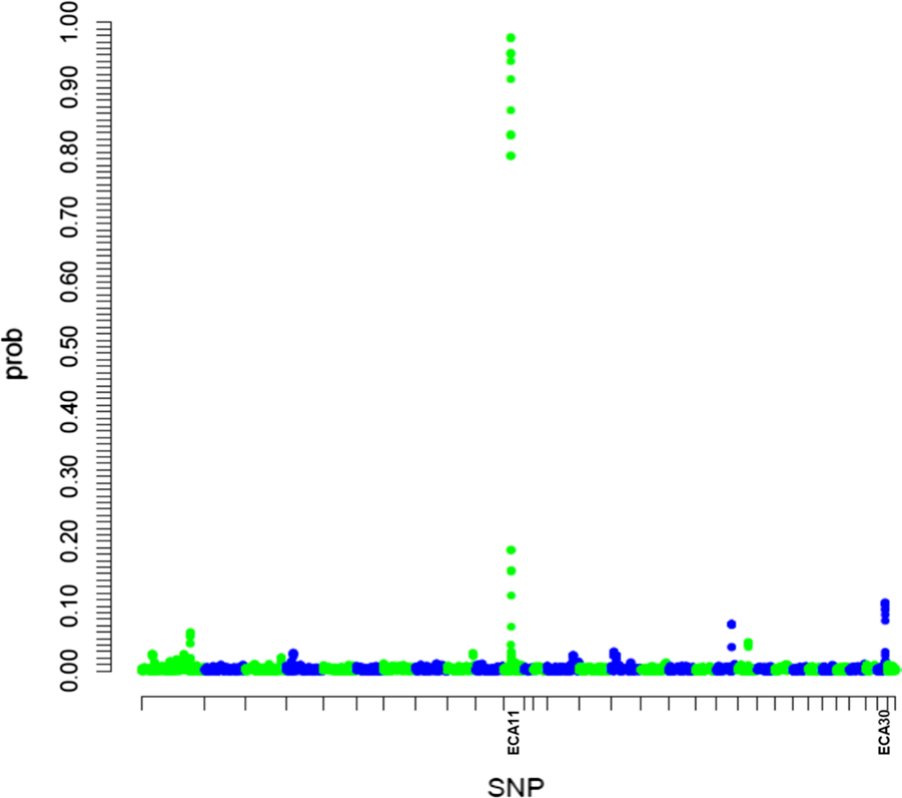
Plot of posterior inclusion probabilities showing a strong signal on ECA11. A genome-wide association study based on 46,215 SNPs was performed using a Bayesian sparse linear mixed model (BSLMM). Cumulative inclusion probabilities were computed for sliding windows of 15 SNPs and were plotted along the genome, taking the central SNP as reference. These data represent the probability for a region to have an effect above the polygenic background. A single strong signal encompassing the *type I keratin* gene cluster was observed on ECA11, with seven windows showing inclusion probabilities above 75% and an additional weak signal on ECA30 was also detected (four windows with inclusion probabilities under 20%)

In the absence of reliable information on the inheritance of curly phenotype in the population studied, we decided to perform a GWAS. A Bayesian sparse linear mixed model (BSLMM) [9] was applied to the Illumina EquineSNP50 BeadChip genotyping data of 51 curly and 19 straight-haired French and North American horses from 13 different paternal families (see “Methods” section). This hybrid GWAS analysis approach combines a linear mixed model and a sparse regression model using Markov chain Monte Carlo (MCMC) to identify associated SNPs by jointly modeling all SNPs while controlling for population structure. BSLMM was shown to yield good performance across a wide range of scenarios, including datasets with a small number of samples. A strong signal that encompassed the *type I keratin* gene cluster was observed on ECA11 (Fig. 2), with seven sliding windows of 15 SNPs showing cumulative inclusion probabilities higher than 75%. Other additional weak signals were observed, including four windows with probabilities lower than 20% on ECA30.

### Haplotype and pedigree analysis support genetic heterogeneity of the curly hair phenotype

To gain further insight into the localization of the locus, haplotypes of 21 SNPs covering the mapping interval were reconstructed based on pedigree information, ranging from positions 21,357,111 to 22,715,359 bp on the EquCab2.0 genome assembly [8] (see Additional file 1: Table S2a). Haplotypes were mined to identify a putative identical-by-descent segment shared by the curly animals and to define the critical Crd mapping region (see Additional file 1: Table S2b). A unique haplotype of ten SNPs (GGAGAGAAAA) between SNP BIEC2-143610 (21,865,941 bp) and SNP BIEC2-143955 (22,699,231 bp) was observed in 46 out of the 51 curly animals, whereas it was absent from the 19 straight-haired animals (chi_square p value = 1.5 × 10^−12^). Among these 46 animals, 35 were heterozygous and 11 were homozygous for this haplotype, which supports a dominant genetic inheritance for the phenotype associated with this haplotype (Table 1).

**Table 1:** Haplotype counts in the critical mapping region (SNP names and chromosome positions are in Additional file 1: Table S2a)

Haplotype	Curly animals	Straight-haired animals					Total
	Number of haplotypes	Heterozygous state	Homozygous state	Number of haplotypes	Heterozygous state	Homozygous state	
GGAGAGAAAA	57	35	22	0	0	0	57
AGAGGGGCGG	12	12	0	17	9	8	29
AGAGGGAAAA	6	6	0	3	3	0	9
AAAGGAAAAA	3	3	0	2	2	0	5
AGAGAGGCGG	3	3	0	2	2	0	5
AGAGGGAAAG	3	3	0	2	2	0	5
GGAGAAACGA	4	4	0	0	0	0	4
AGAAGAGCGG	3	3	0	1	1	0	4
AAAGGGAAAA	2	2	0	1	1	0	3
AGAAAGACGG	1	1	0	2	2	0	3
GGAGAGGCGG	0	0	0	2	2	0	2
AGAGAAAAAA	0	0	0	2	2	0	2
AGAGAGAAAA	2	2	0	0	0	0	2
AGAGGAAAAG	1	1	0	1	1	0	2
AGAGGAACGA	1	1	0	1	1	0	2
AGGAGAGCGG	2	2	0	0	0	0	2
Four rare haplotypes	2	2	0	2	2	0	4
Total	102	80	22	38	33	5	140

Interestingly, the five curly horses that do not carry haplotype GGAGAGAAAA share a common curled ancestor about six to eight generations back. In contrast to most families included in this study, which are related to the Damele stallion line, these horses descend by both their parents from Walker’s Prince T, a stallion registered in both the Missouri Fox Trotter and Curly Horse registries.

### Whole-genome sequencing identifies a missense variant in the *keratin 25* gene as candidate causal variant for a dominant curly hair phenotype

**Fig. 3:**
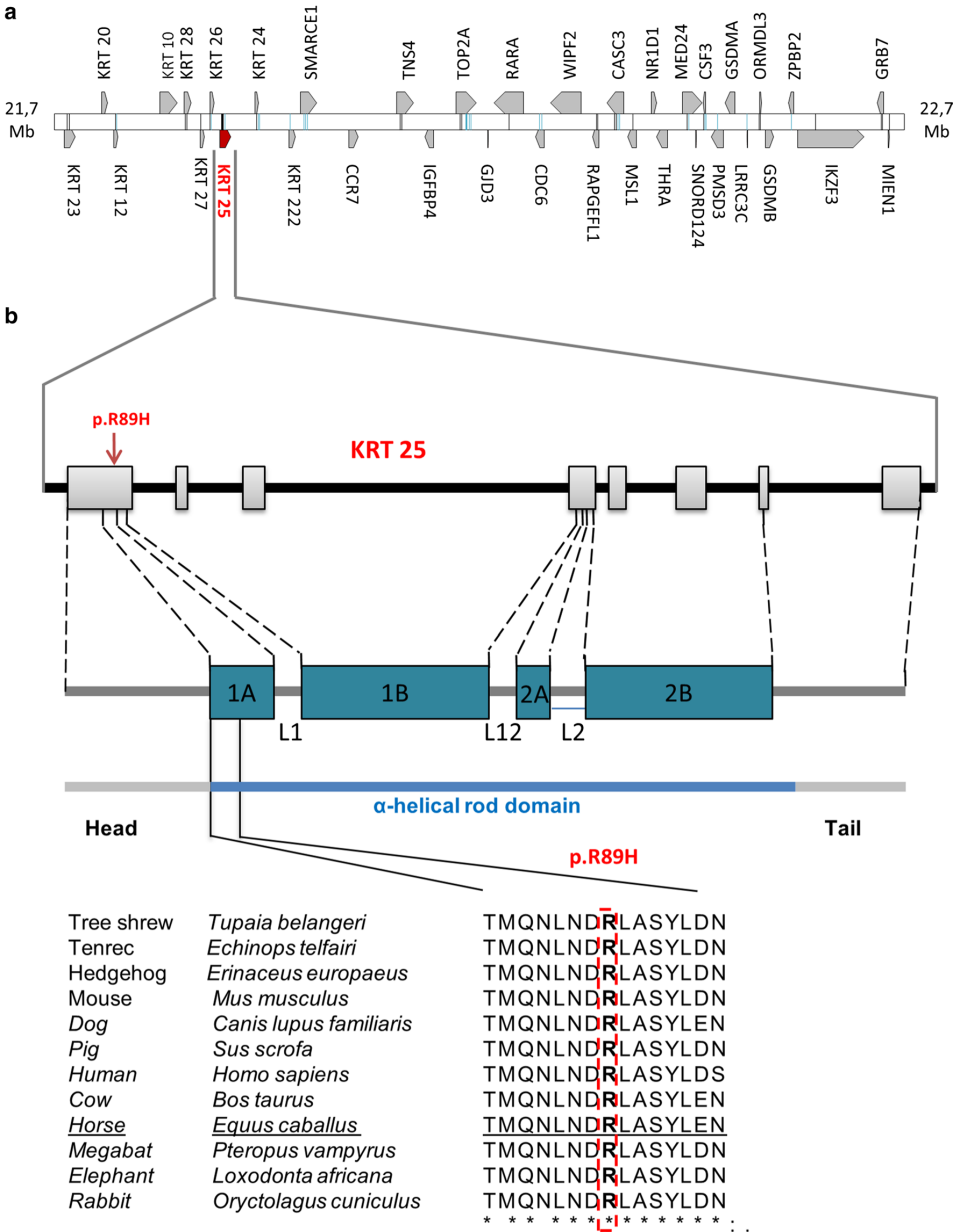
Fine-mapping of the *Crd* locus in horse and whole-genome sequencing identify a candidate variant in the coil1A domain of *KRT25* that affects an amino-acid residue conserved among placental mammals. **a** The IBD segment delineated by haplotype analysis comprises 36 genes, including 10 genes encoding keratin proteins (*KRT-10*, *-12*, *-20*, *-23*, *-24*, *-25*, *-26*, *-27*, *-28*, *-222*). **b** Multispecies alignment of *KRT25* orthologues shows a perfect conservation of amino acid R89. The p.R89H variant affects the coil1A domain within the α-helical rod domain of KRT25 protein. Ensembl accession numbers for the wild type transcript of each species are ENSTBEP00000001955, ENSETEP00000006824, ENSEEUP00000013767, ENSMUSP00000048439, ENSCAFP00000032235, ENSSSCP00000018507, ENSP00000310573, ENSBTAP00000040707, ENSECAP00000011587, ENSPVAP00000010713, ENSLAFP00000005738 and ENSOCUP00000007766 in order of appearance

The Crd IBD segment comprises 36 genes (Fig. 3a) including 10 keratin coding genes (*KRT*-*10*, -*12*, -*20*, -*23*, -*2*4, -*25*, -*26*, -*27*, -*28*, -*222*).

To comprehensively identify candidate causal variants within these genes, the complete genomes of a heterozygous curly horse for haplotype GGAGAGAAAA (BCF SPARTACULAR SPLASHES) and its straight-haired son (JAK ALIAS SPLASH) were sequenced using 100-bp paired-end reads. About 370 million reads were obtained and 360 million reads mapped to the EquCab2.0 genome assembly (36 Gb of mapped sequences). Among these, 90% passed the quality filtering step (score less than 30) and 22% were discarded based either on mapping uniqueness or redundancy after GATK realignment (to remove duplicates). This stringent filtering procedure was applied to reduce bias and improve SNP reliability. As a result, about 65% of the reads were retained, leading to a 9X mean genomic coverage.

About 7,200,000 SNPs were detected, including 21,680 SNPs located in 10,867 genes and predicted to impact protein functionality (non-synonymous, nonsense or stop loss, based on Ensembl variant effect predictor (see Additional file 1: Tables S4 and S5).

The genotypes of the two sequenced animals agreed with their status at the Crd locus (i.e. curly animal heterozygous and straight-haired animal homozygous for the reference allele) for about 900,000 SNPs which were also absent from dbSNP. Among these, 2482 were predicted to impact protein functionality. Within the curly interval, 452 positional candidate SNPs were identified (see Additional file 1: Table S6), including four non-synonymous variants (g.21891160G>A, g.21932167G>T, g.22186465C>T, g.22191762G>T,) i.e. KRT25:p.R89H, KRT24:p.G51C, TOP2A:p.S1088L, TOP2A:p.L1284F). Table 2 provides detailed information on these four non-synonymous candidate SNPs.

**Table 2:** Details on ECA11 candidate non-synonymous SNPs

Position (bp)	Ref. allele	Alternate allele	Gene	Protein accession number	Consequence	SIFT
21,891,160	G	A	*KRT25*	ENSECAP00000011587	p.R89H	Deleterious p = 0.01
21,932,167	G	T	*KRT24*	ENSECAP00000013915	p.G51C	Tolerated p = 0.05
22,186,465	C	T	*TOP2A*	ENSECAP00000011684	p.S1088L	Deleterious p = 0.05
22,191,762	G	T	*TOP2A*	ENSECAP00000011684	p.L1284F	Deleterious p = 0.01

**Fig. 4:**
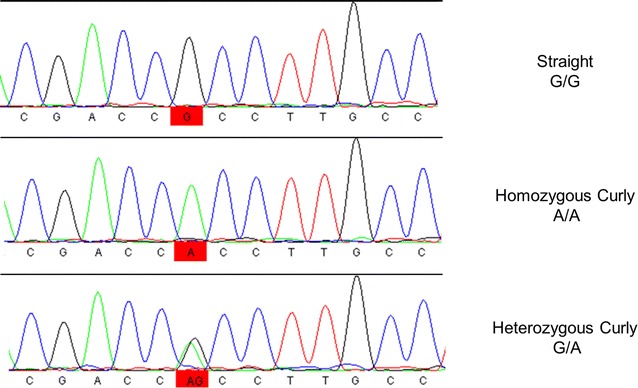
Chromatograms showing the g.21891160G>A variant within the *KRT25* gene on ECA11

Sanger sequencing was performed on PCR fragments to confirm the existence of these SNPs (Fig. 4).

A subset of 24 samples representative of the diverse haplotypes was analysed to infer genotypes for the whole mapping panel (see Additional file 1: Table S7). An additional set of 16 samples was also genotyped, including five horses related to the discordant individuals. As a result, it was proven that SNPs KRT24:g.21932167G>T and Top2A: g.22191762G>T were carried by straight-haired horses and could thus be discarded. In addition, 65% of the genotyped curly horses were found to be homozygous for the reference allele (*CC*), thus the causality of the Top2A:g.22186465C>T SNP was very unlikely. Genetic association with KRT25:g.21891160G>A was then confirmed by genotyping a larger population consisting of 100 horses registered as curly, 50 horses registered as straight-haired as well as 203 controls from a diverse set of breeds. No straight-haired horse carried allele *A*. However, no complete association was observed among curly animals. Indeed, the five discordant curly horses that do not carry the main associated haplotype and all their relatives had the *GG* genotype (see Additional file 1: Table S7), which suggested genetic heterogeneity. Thus, SNP KRT25:p.R89H may be causal, but a second locus or allele may also be involved in shaping the hairs within North American Curly horses. The *KRT25* sequence was established using whole-genome sequencing data from two discordant horses to explore a putative allelic heterogeneity. No other heterozygous deleterious SNP was identified, thus, the hypothesis of a second dominant SNP in the same gene was excluded (see Additional file 1: Table S6). One could speculate on the existence of a recessive SNP of Missouri Fox Trotter origin since discordant curly horses trace back to Walker’s Prince T, a curled stallion, who was registered in both the Missouri Fox Trotter and Curly Horse registries. Walker’s Prince T was the product of the multigenerational crossing of Curly Horses with Missouri Fox Trotters. However, the curly phenotype can be excluded as a recessive mutation of Missouri Fox Trotter origin, since the pedigree shows a dominant mode of transmission stemming from a dominant curly stallion of unknown origin named Curly Jim. In addition, Dravkvallons Ite O Maguzu, a discordant stallion related to Walker’s Prince T, was found to be heterozygous for a dominant curly gene, since it gave birth to curly and straight-haired horses when crossed with dams from a non-curly breed (see Additional file 1: Table S8). Consequently, our data suggest that a second dominant locus that is located away from the current region could be responsible for the curly phenotype in certain Curly, as well as Curly crossed with Missouri Fox Trotter lines. This hypothesis is consistent with some phenotypic features observed in such homozygous horses but not with phenotypes in horses homozygous for the mutated *KRT25* allele, such as tight curls on the body guard hairs known as the “brillo-pad coat”, as well as shorter (but not sparse) hairs on manes and tails.

We cannot formally exclude the possibility that we missed the true causal variant due to mis-assembly or incorrect annotations of the reference genome, but we believe that it is very unlikely. While the horse genome assembly and its annotation are still imperfect, we did not note major discrepancies between the horse, human (GRCh38.p10) and mouse (GRCm38.p5) assemblies that are available in Ensembl regarding the number and the nature of keratin coding genes located within the critical mapping interval. The synteny is perfectly preserved, sequence gaps cover intergenic regions and no keratin coding gene is missing in this region in the horse genome assembly. A comparison of the NCBI and Ensembl horse annotations revealed missing predicted keratin-associated protein genes in the Ensembl database. However, these genes are located upstream of the critical mapping region (from ECA11: 21,369,668 bp to ECA11: 21,659,203 bp, see Additional file 1: Table S9). No discrepancy was noted within the critical region, between *KRT40* and *TOP2A*, except some differences in terms of gene start or stop due to differences in the 5′UTR and 3′UTR lengths.

As a further verification, we mined the Mouse Genome Informatics database in an attempt to identify other functional candidate genes within the mapping interval (http://www.informatics.jax.org/). In addition to *KRT25*, only *KRT27* was previously shown to affect hair growth and morphology, with homozygous mutants for different variants presenting wavy coat or rex and wavy coat [17]. Finally, we used the Integrative Genome Viewer to check for the presence or absence of putative structural variants around these two candidate genes. We did not find any large insertion or deletion involving intronic or exonic portions of these genes. This was expected since large insertion–deletions, duplications or inversions have dramatic consequences on the affected genes and resulting proteins while the curly phenotype is usually due to substitutions or small insertion–deletions of a multiple of three bases affecting the coil domains of keratin proteins.

### Presumed consequences of the KRT25:p.R89H variant

SNP g.21891160G>A (KRT25:p.R89H) affects the coil1A domain of KRT25, a type I inner root sheath-specific keratin that is essential for the proper assembly of type I and type II keratin protein complexes and the formation of keratin intermediate filaments in the inner root sheath of hair. Interestingly, several SNPs that affect the α-helical rod domains of different keratins have been associated with wavy or curly phenotypes in humans (*KRT25*, *KRT74*) [18, 19], mice (*KRT25*, *KRT27*) [20], dog (*KRT71*) [21], cat (*KRT71*) [22, 23] and cattle (*KRT27*) [24]. Furthermore, two other SNPs in the coil1 domain (http://mutagenetix.utsouthwestern.edu/home.cfm) and coil2 domain of *KRT25* [20] were previously reported to be responsible for dominant hair phenotype in mice (Plush and Sinuous, as well as Re and M100573 mice).

The KRT25:p.R89H horse variant is predicted to be damaging by POLYPHEN and SIFT software [25, 26]. In addition, multispecies alignment of *KRT2*5 orthologs (Fig. 3b) and horse paralogs (not shown) show a perfect conservation of amino acid R89, which indicates that this amino acid plays an essential role not only in *KRT25* but also in type I keratin function. Interestingly, while missense variants in these genes have been associated with curly phenotypes, more deleterious variants such as nonsense, frameshift, splicing defects or deletions are responsible for more severe phenotypes such as Rex wavy coat (Re^wc^) in mouse [20] and Devon Rex or hypotrichosis (Sphynx breed) in cat [22]. Taken together these arguments strongly support a causative role for KRT25:p.R89H in the dominant curly phenotype observed in the North American Curly horse breed.

Finally, our study also provides evidence for genetic heterogeneity. Thus, it is tempting to speculate that the range of diverse and seemingly complex phenotypes observed in curly horses may result from a combinatorial effect of several genes. Such a hypothesis is already validated in dogs, where most coat variation in 108 modern domestic dog breeds can be explained by the combination of three variants in the *RSPO2*, *FGF5* and *KRT71* genes [21].

## Conclusions

We report the identification of the first curly variant in horse and suggest that a second dominant locus also segregates in the population, in contrast to the current hypotheses. On the one hand, the identified variant will help breeders to optimize their mating designs to produce curly horses. On the other hand, ascertained status for the *KRT25* variant will also make the search for other loci easier. Further studies are required to unravel the complete genetic determinism, elucidate the role of *KRT25* domains in hair follicle morphogenesis and hair growth, as well as to explore the link between hair structure and hypoallergenic of curly horses.

## Additional file

**Additional file 1: Table S1.** List of animals included in the study. This table provides detailed information about identification, pedigree, phenotypes and genotypes of animals included in the study. The full pedigree is also provided. **Table S2a.** Details on markers and haplotypes in the mapped region. This table provides markers and their position, including the *KRT25* variant along ECA11. Colors are used to depict haplotypes, assuming 18 founder haplotypes (12 “non-curly” haplotypes carried by crossbreed animals as well as the six most frequent other haplotypes, including one “curly” haplotype). Haplotypes are given for each animal, according to pedigree data. **Table S2b.** Delineation of the critical mapping region by haplotype analysis. Haplotypes were sorted out according to their sequence and the associated coat phenotype. Critical recombination events are easily detected by color changes and help identify the upper and lower bounds of the mapping interval. **Table S3.** PCR primers used to genotype candidate genes. This table provides detailed information about chromosome positions, alleles, gene names, ID, primer sequences, fragment lengths and Tm. **Table S4.** Statistics on genome-wide detection of functional variants. Table S4 provides statistics on variant detection based on NGS sequencing, with numbers of new and known variants (whole genome and genes) according to their consequence as predicted by VEP. Positional candidate variants are also reported. **Table S5.** List of gene variants predicted to impact protein functionality. Table S5 includes chromosome positions, alleles, gene ID and functional annotations. The last column (Existing variation) indicates variants; which were already known in dbSNP. **Table S6.** Concordant variants identified in the critical mapping interval. Table S6 includes chromosome positions, alleles, functional annotations and gene ID for concordant variants. **Table S7.** Horses genotyped by PCR and Sanger sequencing. Horse names, phenotype and genotype for the KRT24:g.21932167G>T, Top2A:g.22186465C>T and Top2A: g.22191762G>T variants are presented. **Table S8.** Pedigree information for the progeny of Walker’s Prince T and Dravkvallons Ite O Maguzu. Table S8 provides pedigree and phenotype data obtained from breeders and the Curly horse pedigree database (http://www.curlyhorses.info/mainsearch.asp). **Table S9.** Comparison of NCBI and Ensembl annotations within the keratin cluster on ECA11. Annotation features are listed along ECA11, from position 2,136,900 to 2,197,100 pb; some discrepancy is observed upstream of the critical mapping interval.
